# Diagnostic Overlap between Fanconi Anemia and the Cohesinopathies: Roberts Syndrome and Warsaw Breakage Syndrome

**DOI:** 10.1155/2010/565268

**Published:** 2010-07-18

**Authors:** Petra van der Lelij, Anneke B. Oostra, Martin A. Rooimans, Hans Joenje, Johan P. de Winter

**Affiliations:** Department of Clinical Genetics, VU University Medical Center, Van der Boechorststraat 7, 1081 BT Amsterdam, The Netherlands

## Abstract

Fanconi anemia (FA) is a recessively inherited disease characterized by multiple symptoms including growth retardation, skeletal abnormalities, and bone marrow failure. The FA diagnosis is complicated due to the fact that the clinical manifestations are both diverse and variable. A chromosomal breakage test using a DNA cross-linking agent, in which cells from an FA patient typically exhibit an extraordinarily sensitive response, has been considered the gold standard for the ultimate diagnosis of FA. In the majority of FA patients the test results are unambiguous, although in some cases the presence of hematopoietic mosaicism may complicate interpretation of the data. However, some diagnostic overlap with other syndromes has previously been noted in cases with Nijmegen breakage syndrome. Here we present results showing that misdiagnosis may also occur with patients suffering from two of the three currently known cohesinopathies, that is, Roberts syndrome (RBS) and Warsaw breakage syndrome (WABS). This complication may be avoided by scoring metaphase chromosomes—in addition to chromosomal breakage—for spontaneously occurring premature centromere division, which is characteristic for RBS and WABS, but not for FA.

## 1. Introduction

Fanconi anemia (FA) is a recessive chromosomal instability syndrome that is clinically characterized by a wide variety of symptoms including growth retardation and developmental abnormalities such as malformed digits, absent radii, and microcephaly. Additional common features include a progressive bone marrow failure and pronounced cancer predisposition. Because the FA phenotype is so diverse and variable, diagnosis on the basis of clinical features alone is often difficult [1–3]. 

The ultimate diagnosis of FA has been based on a hyperresponsiveness of FA cells to chromosome breakage by DNA cross-linking agents, such as diepoxybutane (DEB) and mitomycin C (MMC), or on excessive cell cycle arrest in the G2/M phase of the cell cycle, both spontaneously and after treatment with MMC [4–6]. In a number of patients, spontaneous genetic reversion can correct FA mutations in haematopoietic stem cells, leading to mosaicism in the blood. The reverted cells may (partially) correct the bone marrow failure [7–11]. In mosaic FA patients, the overall cross-linker hypersensitivity is less pronounced, because in the blood of such patients phenotypically normal cells exist in addition to FA cells. As genetic reversion has not been observed in tissues other than blood, performing the test on the patient's skin fibroblasts helps to avoid this complication. 

An occasional patient suffering from Nijmegen breakage syndrome or some hitherto undefined disorder has been noted to score positive in the FA chromosomal breakage test [12–14]. Cellular hypersensitivity to MMC has also been reported for a distinct class of syndromes, the so-called “cohesinopathies”. These are caused by mutations in genes involved in the process of sister chromatid cohesion [15] and include Cornelia de Lange syndrome, Roberts syndrome [16–18], and the recently described Warsaw Breakage syndrome [19]. Due to a highly variable clinical phenotype, Roberts syndrome (RBS) patients may exhibit symptoms overlapping with those of FA. RBS is an extremely rare, autosomal recessive disorder characterized by severe pre- and postnatal growth retardation, craniofacial abnormalities, and symmetric limb defects, features that are also observed in FA. RBS is caused by mutations in *ESCO2*, which encodes an acetyltransferase that is involved in sister chromatid cohesion [20]. Affected individuals show varying degrees of malformations involving symmetric reduction in the number of digits and the length or presence of bones in the arms and legs [21]. So far, no clear correlation between the type of mutation and clinical phenotype has been observed [22–24]. Most cases of RBS result in spontaneous abortion, stillbirth, or neonatal death; mental retardation is often observed, with various degrees of severity. At the cellular level, RBS patients show specific cytogenetic features, mainly consisting of metaphase chromosomes displaying repulsion of heterochromatic regions, leading to a railroad-track appearance of the chromosomes. This cytogenetic feature is used in the diagnosis of RBS and may be followed by mutational analysis of *ESCO2*.

Recently, an individual was described with mutations in another gene involved in sister chromatid cohesion, *DDX11/ChlR1*, which encodes an XPD-like DNA helicase. This novel cohesinopathy, called Warsaw breakage syndrome (WABS), is mainly characterized by severe growth retardation and microcephaly [19]. The patient representing this syndrome was initially suspected to suffer from a chromosomal instability syndrome and was therefore tested for chromosomal breakage in an FA-specific diagnostic test. Metaphase preparations revealed cohesion defects (as in RBS), that is, railroad-track appearance of chromosomes due to premature centromere division (PCD); in addition to this feature, a high proportion of metaphases showed premature chromatid separation (PCS), in particular after treatment with MMC.

Here we report that patients suffering from RBS or WABS may be misdiagnosed as having FA based on excessive MMC-induced chromosomal breakage in primary lymphocyte cultures and/or on an increased arrest of primary skin fibroblasts in the G2/M phase of the cell cycle. However, detailed cytogenetic analysis of unchallenged lymphocyte cultures should reveal the presence of railroad track-like chromosomes in RBS and WABS, which are not known to occur in unchallenged FA lymphocyte cultures. We conclude that in the diagnostic work-up for FA, cytogenetic examination should include the scoring of cohesion defects in unbanded metaphase preparations, in order to exclude the cohesinopathies RBS and WABS.

## 2. Methods

### 2.1. Patients

All affected individuals investigated (Table 1) have been reported before. RBS patients with homozygous mutations in *ESCO2* and heterozygous carriers (mothers) were as follows: VU1174 (c.1457_1458delAG) (Turkey); VU1199 (c.877_878delAG) (Turkey) and VU1200 (mother of VU1199); VU1366 (c.762_763delTT) (Tunisia); VU1400 (c.877_878delAG) (The Netherlands) and VU1401 (mother of VU1400) [20, 23, 24]; and WABS patient VU1202 with biallelic mutations in *DDX11* (IVS22+2C>T and c.2689_2691delAAG) and his mother (VU1203, IVS22+2C>T) [19]. The FA-J patient EUFA1333 and FA-B patient EUFA1386 were reported before [25, 26]. Three healthy individuals (laboratory personnel, ML, NN, and Bebu) served as controls in the cytogenetic analyses.

### 2.2. Lymphocyte Chromosomal Breakage Test

Freshly drawn heparinized venous blood (≥2 mL) was used to prepare whole-blood cultures as usual for routine cytogenetic analysis. Next to two healthy controls, blood samples were analyzed from the RBS patients (RBS-1, -2, and -3), heterozygous mothers (Con-1 and -2), the WABS patient (WABS) and his heterozygous mother (Con-3), FA-B patient FA-2, and the mosaic FA-J patient FA-1. Four cultures per individual were prepared by adding 0.5 mL blood to 4.5 mL complete medium (RPMI (Gibco, Grand Island NY, USA) including 15% fetal bovine serum (Hyclone, Logan, USA) streptomycin, penicillin (both Gibco), and phytohemagglutinin (PHA) as prescribed (Remel inc., Lenexa, USA). The whole-blood cultures were incubated for 72 h with 0, 50, 150, or 300 nM mitomycin C (MMC; Kyowa Hakko Kogyo Co., Tokyo, Japan). After treatment with demecolcin (Sigma, St. Louis, USA, 200 ng/mL) for 40 minutes, cells were harvested, incubated with 0.075 M KCL for 20 minutes at room temperature, and fixed with 75% methanol, 25% acetic acid. Cells were dropped on a microscope slide and stained for 5 minutes in a 5% Giemsa solution (Merck); no banding technique was applied. For each culture, 50 metaphases were examined for chromosomal breakage and cohesion defects, on coded slides. The presence of chromatid breaks and interchanges was expressed as break events per cell, counting chromatid interchange figures as the minimum number of break events required for their reconstruction [27]. Metaphases showing cohesion defects were recorded in one of five categories: (1) 1-2 railroad chromosomes (“railroads”) per cell, (2) 3–5 railroads per cell, (3) >5 railroads per cell, (4) >5 railroads plus one or more chromosomes with total chromatid separation, (5) total premature chromatid separation (PCS); for illustrations, see Figure 3(b).

### 2.3. Cell Cycle Analysis of Primary Fibroblasts

Primary skin fibroblasts from two RBS patients (RBS-1 and -4), the WABS patient, two FA patients (FA-1 and -2), and a healthy control were cultured for 72 hours either without or with MMC (50 or 100 nM) in Ham's F10 medium (Gibco, Paisley, UK) supplemented with 10% fetal bovine serum (Hyclone). Cells were harvested by trypsinization and permeabilized in buffer containing 100 mM Tris-HCL (pH 7.5), 150 mM NaCl, 0.5 mM MgCl_2_, 1 mM CaCl_2_, 0.2% BSA, and 0.1% NP-40, followed by staining of DNA with PI/RNase staining buffer (BD Pharmingen, BD Biosciences, San Jose, USA). Cell suspensions were analyzed by flow cytometry on a BD FACScalibur (BD Biosciences), to determine G2/M accumulation as the percentage of cells present in the G2/M phase of the cell cycle. 

## 3. Results

### 3.1. Diagnostic Overlap of FA with RBS in a Chromosomal Breakage Test

**Figure 1:**
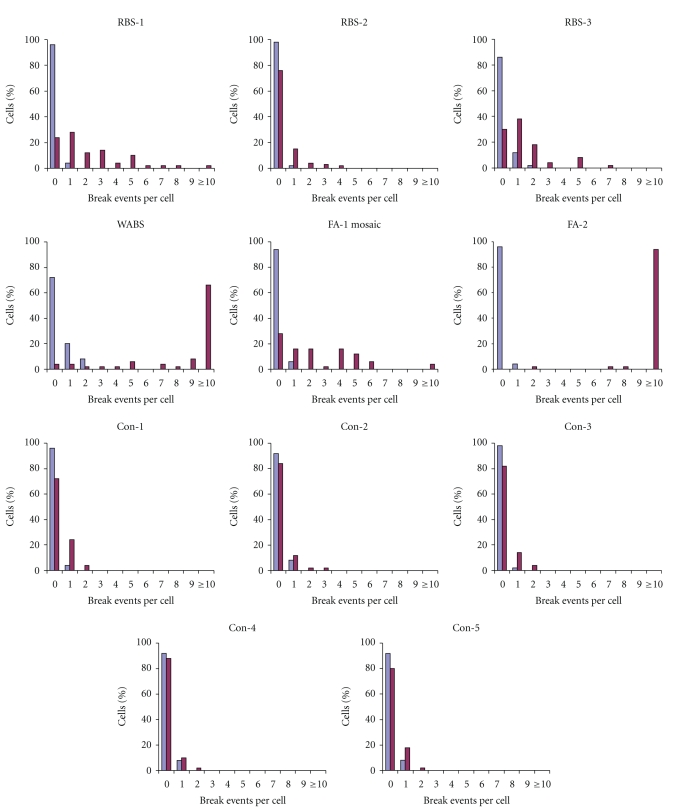
Chromosomal breakage in stimulated T lymphocytes from patients with FA, RBS, or WABS. A chromosomal breakage test, routinely utilized for the diagnosis of FA, was carried out on various individuals, as indicated (RBS: Roberts syndrome; WABS: Warsaw Breakage syndrome; FA: Fanconi anemia: FA mosaic, FA patient with hematopoietic mosaicism; Con-1 to -3: healthy controls, that is, parents of an affected individual; Con-4 and -5: healthy controls, noncarriers; see Table 1). Whole-blood PHA-stimulated T lymphocyte cultures were exposed to various concentrations of MMC for 72 h and analyzed for chromosomal breakage. Only results for the untreated (blue bars) and the highest concentration of MMC (300 nM, purple bars) are shown. Percentages of cells with the indicated number of chromatid-type break events per cell are shown.

Three RBS patients were included in an FA-specific diagnostic test and examined for chromosomal breakage in PHA-stimulated T lymphocytes. In this test, lymphocytes of RBS patients RBS-1 and -3 revealed an MMC-induced excessive increase in chromosomal breakage events when compared to a healthy control (Figure 1). No evidence for an excessive chromosomal breakage rate was found in patient RBS-2. The mean overall breakage rates (break events per cell) after treatment with the highest MMC concentration (300 nM) were 2.22 in RBS-1, 0.04 in RBS-2, and 1.4 in RBS-3. Compared to an average of 2.54 break events per cell in the mosaic FA-J patient (FA-1) and 0.14–0.22 in the healthy controls, patients RBS-1 and -3 appeared to fall in the breakage range of mosaic FA patients. In addition, mono-allelic *ESCO2* mutation carriers (mothers of patients RBS-1 and -3) did not reveal significantly increased chromosomal breakage rates (Con-1 and -2, Figure 1). Mutational analysis revealed a homozygous c.877delAG mutation in exon 4 of the *ESCO2* gene in both RBS-1 and -3, which was considered remarkable since these individuals were from unrelated ethnic backgrounds (Turkish and Dutch). Patient RBS-2 had a c.762delTT mutation in exon 3b.

### 3.2. Diagnostic Overlap of FA with WABS in a Chromosomal Breakage Test

The WABS patient was tested for FA, after he had been excluded from ataxia telangiectasia, Nijmegen breakage syndrome, and Bloom syndrome [19]. At 150 nM MMC the number of break events in WABS cells was clearly elevated compared to a healthy control, but somewhat less pronounced than in FA (data not shown). At the highest concentration (300 nM) the average breakage rate was 8.28 break events/cell, which falls in the range typical for full-blown FA (Figure 1).

### 3.3. Diagnostic Overlap of FA with RBS and WABS in a Cell Cycle Test

**Figure 2:**
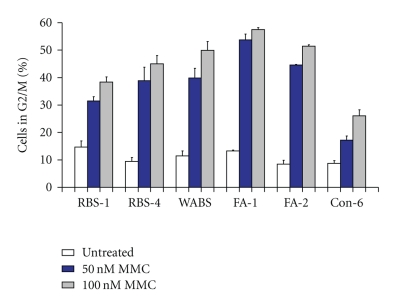
Cell cycle analysis of primary skin fibroblasts from individuals with FA, RBS, or WABS. Cells were treated with 50 or 100 nM MMC, for 72 h. Cell cycle profiles were obtained and the percentages of cells in G2/M phase of the cell cycle were determined. Averages of at least two experiments are shown, with standard errors of the mean.

Besides the chromosomal breakage test, the FA diagnosis may also be based upon the increased arrest of FA cells in G2/M phase of the cell cycle upon treatment with MMC [4–6]. In this test, primary skin fibroblasts are exposed to MMC and analyzed by flow cytometry for the percentage of cells in the G2/M phase of the cell cycle. We treated primary skin fibroblasts from RBS patients RBS-1 and -4 and the WABS individual with MMC (50 or 100 nM) and analyzed their cell cycle distribution after 72 hours of drug exposure. In line with the results of the chromosomal breakage test, both RBS and WABS fibroblasts showed an increased response to MMC in terms of the extent of their accumulation in G2/M compared to fibroblasts from a healthy control individual (Figure 2). Concordant with the chromosomal breakage test, the response to MMC in the WABS patient was similar to that in FA (approximately 50% of the cells in G2/M at 100 nM MMC). The least pronounced response was observed in RBS patient RBS-1 (38% in G2/M, Figure 2), which seemed to correlate with the moderate breakage rate observed in the chromosomal breakage test. However, as illustrated by patient RBS-4, some cases with RBS may behave as a genuine FA case in a cell cycle analysis test (48% in G2/M; Figure 2). 

### 3.4. Cohesion Defects Distinguish RBS and WABS from FA

**Figure 3:**
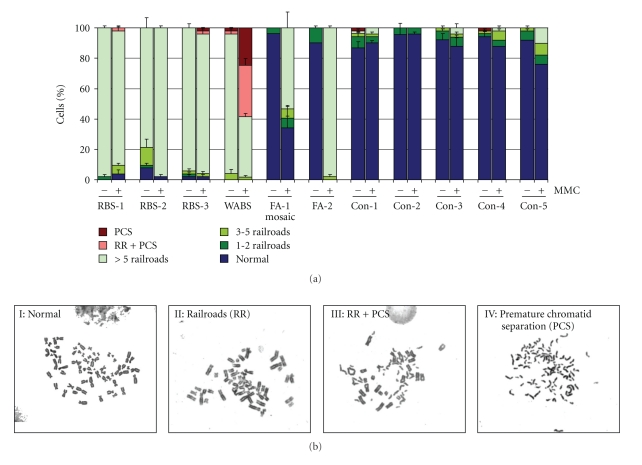
(a) Cohesion defects in lymphocyte cultures from patients with FA, RBS, or WABS. Metaphases prepared from untreated cultures (−) or treated with 150 nM MMC (+) which had previously been evaluated for chromosomal breakage were reevaluated for the presence of cohesion defects. The percentages of cells containing the indicated number of railroad chromosomes (RR) or total premature centromere separation (PCS) were determined, as summarized in the histograms. (b) Illustrations of the various aberrations scored in the analysis.

Next to the increased chromosomal breakage and G2/M arrest after MMC treatment in RBS and WABS cells, another clear phenotype was seen at the chromosomal level. Metaphases of the primary lymphocyte cultures showed a drastic increase in the occurrence of cohesion defects (railroad-track chromosomes) in the untreated RBS and WABS patient cells, which were not found in the heterozygous carriers, healthy controls, FA, and FA mosaic patients (Figure 3(a)). The RBS and WABS patients revealed the presence of >5 railroad chromosomes in 79%–98% of the metaphases, while in the controls, heterozygotes, and FA cells this percentage did not exceed 2% of the cells. In addition to the railroad chromosomes cells from the WABS patient showed a massive increase in the total premature sister chromatid separation in response to treatment with MMC at 150 nM. Although untreated FA lymphocyte cultures had normal chromatid cohesion, MMC treatment resulted in a significant cohesion defect in FA lymphocyte cultures as well (FA-1 and -2, Figure 3(a)), albeit that this defect became only manifest as railroad chromosomes, without the occurrence of PCS. 

## 4. Discussion

Although hypersensitivity to MMC and DEB in peripheral blood lymphocytes is an accepted diagnostic criterion for FA, this study shows that in addition to Nijmegen breakage syndrome, also patients with RBS or WABS cells may score positive in an FA-specific MMC-induced chromosomal breakage test or in an MMC-induced G2/M arrest analysis. These affected individuals might have been diagnosed as FA patients (with hematopoietic mosaicism) on the basis of their clinical characteristics in combination with their hypersensitivity to MMC. However, detailed inspection of metaphases for chromosomal cohesion defects and subsequent mutational analysis of the *ESCO2* or *DDX11* genes would have demonstrated that these patients were to be diagnosed as RBS or WABS, respectively.

Next to RBS and WABS, a third cohesinopathy has been known, called Cornelia de Lange syndrome (CdLS). This syndrome is caused by mutations in cohesin complex components (SMC1A and SMC3) or its regulatory factor NIPBL [28–31]. CdLS is a multisystem developmental disorder with classical features of characteristic facial dysmorphisms, upper limb malformations, hirsutism, and growth and cognitive retardation displaying a wide spectrum of clinical severity. Studies have revealed an increased sensitivity to MMC for both fibroblasts and B lymphoblastoid cells of CdLS patients [17], which indicates that CdLS patients may also score positive in an FA-specific diagnostic test leading to a possible diagnostic overlap with this cohesinopathy as well. However, due to the quite characteristic facial appearance of CdLS patients, the chance for a CdLS patient being misdiagnosed as FA seems very small. 

Interestingly, both RBS patients with a c.877delAG mutation showed hypersensitivity for chromosomal breakage by MMC, while the patient with a different mutation had no such hypersensitivity. All *ESCO2* mutations found until now in RBS patients have appeared to affect the acetyltransferase activity of the protein [23]. Patient RBS-1 was strongly affected, with severely shortened arms and legs and a cleft lip. Despite the fact that the patients carried the same mutations (without evidence for a common ancestry), the clinical phenotype of patient RBS-3 was much milder. In this case, patient RBS-3 might have been suspected to have FA, and in combination with the increased MMC sensitivity could have been misdiagnosed as FA. These observations also indicate that there is no obvious correlation between the severity of disease symptoms and the extent of sensitivity to DNA cross-linking agents and support the notion that there is no clear genotype-phenotype correlation in RBS [22].

This report has confirmed that the test for chromosomal hypersensitivity to DNA cross-linking agents is not entirely specific for FA: in addition to Nijmegen Breakage syndrome both RBS and WABS patient cells also show increased sensitivity to MMC. It is therefore to be recommended that in the cytogenetic diagnosis of FA, particularly in clinically atypical patients, quantification of chromosomal cohesion defects is included in the analysis.

In conclusion, although some of the clinical characteristics and the extent of MMC-induced chromosomal breakage in RBS and WABS T lymphocytes show overlap with those of (mosaic) FA patients, inspection of metaphase chromosomes for premature centromere division (PCD, railroad chromosomes) and premature chromatid separation (PCS) allows to distinguish RBS and WABS from FA.

## Figures and Tables

**Table 1:** Individuals studied in this report.

Alias	Patient code	Gene	Mutation(s)	Remarks	ref
RBS-1	VU1199	*ESCO2*	c.877_878delAG	Homozygous	[20, 23, 24]
RBS-2	VU1366	*ESCO2*	c.762_763delTT	Homozygous	[20, 23, 24]
RBS-3	VU1400	*ESCO2*	c.877_878delAG	Homozygous	[20, 23, 24]
RBS-4	VU1174	*ESCO2*	c.1457_1458delAG	Homozygous	[20, 23, 24]
WABS	VU1202	*DDX11*	IVS22+2C>T c.2689_2691delAAG	Compound heterozygous	[19]
FA-1	EUFA1333	*FANCJ*	IVS11–498A>T c.2392C>T	Hematopoietic mosaicism	[25]
FA-2	EUFA1386	*FANCB*	c.811insT	Hemizygous (X-linked)	[26]
Con-1	VU1200	*ESCO2*	c.877_878delAG	Mother of VU1199	
Con-2	VU1401	*ESCO2*	c.877_878delAG	Mother of VU1400	
Con-3	VU1203	*DDX11*	IVS22+2C>T	Mother of VU1202	[19]
Con-4, -5, -6	Healthy controls				

## References

[1] Auerbach AD, Rogatko A, Schroeder-Kurth TM (1989). International Fanconi anemia registry: relation of clinical symptoms to diepoxybutane sensitivity. *Blood*.

[2] Joenje H, Patel KJ (2001). The emerging genetic and molecular basis of Fanconi anaemia. *Nature Reviews Genetics*.

[3] Auerbach AD (2009). Fanconi anemia and its diagnosis. *Mutation Research*.

[4] Kaiser TN, Lojewski A, Dougherty C (1982). Flow cytometric characterization of the response of Fanconi’s anemia cells to mitomycin C treatment. *Cytometry*.

[5] Schindler D, Hoehn H (2007). Fanconi anemia a paradigmatic disease for the understanding of cancer and aging. *Monographs in Human Genetics*.

[6] Pinto FO, Leblanc T, Chamousset D (2009). Diagnosis of Fanconi anemia in patients with bone marrow failure. *Haematologica*.

[7] Kwee ML, Poll EHA, Van de Kamp JJP (1983). Unusual response to bifunctional alkylating agents in a case of Fanconi anaemia. *Human Genetics*.

[8] Lo Ten Foe JR, Kwee ML, Rooimans MA (1997). Somatic mosaicism in Fanconi anemia: molecular basis and clinical significance. *European Journal of Human Genetics*.

[9] Waisfisz Q, Morgan NV, Savino M (1999). Spontaneous functional correction of homozygous Fanconi anaemia alleles reveals novel mechanistic basis for reverse mosaicism. *Nature Genetics*.

[10] Gregory JJ, Wagner JE, Verlander PC (2001). Somatic mosaicism in Fanconi anemia: evidence of genotypic reversion in lymphohematopoietic stem cells. *Proceedings of the National Academy of Sciences of the United States of America*.

[11] Gross M, Hanenberg H, Lobitz S (2002). Reverse mosaicism in Fanconi anemia: natural gene therapy via molecular self-correction. *Cytogenetic and Genome Research*.

[12] Gennery AR, Slatter MA, Bhattacharya A (2004). The clinical and biological overlap between Nijmegen breakage syndrome and Fanconi anemia. *Clinical Immunology*.

[13] New HV, Cale CM, Tischkowitz M (2005). Nijmegen breakage syndrome diagnosed as Fanconi anaemia. *Pediatric Blood and Cancer*.

[14] Bakhshi S, Joenje H, Schindler D (2006). A case report of a patient with microcephaly, facial dysmorphism, mitomycin-c-sensitive lymphocytes, and susceptibility to lymphoma. *Cancer Genetics and Cytogenetics*.

[15] Liu J, Krantz ID (2008). Cohesin and human disease. *Annual Review of Genomics and Human Genetics*.

[16] Van den Berg DJ, Francke U (1993). Sensitivity of Roberts syndrome cells to gamma radiation, mitomycin C, and protein synthesis inhibitors. *Somatic Cell and Molecular Genetics*.

[17] Vrouwe MG, Elghalbzouri-Maghrani E, Meijers M (2007). Increased DNA damage sensitivity of Cornelia de Lange syndrome cells: evidence for impaired recombinational repair. *Human Molecular Genetics*.

[18] van der Lelij P, Godthelp BC, van Zon W (2009). The cellular phenotype of Roberts syndrome fibroblasts as revealed by ectopic expression of ESCO2. *PloS One*.

[19] van der Lelij P, Chrzanowska KH, Godthelp BC (2010). Warsaw Breakage syndrome, a cohesinopathy associated with mutations in the XPD Helicase family member DDX11/ChlR1. *American Journal of Human Genetics*.

[20] Vega H, Waisfisz Q, Gordillo M (2005). Roberts syndrome is caused by mutations in *ESCO2*, a human homolog of yeast *ECO1* that is essential for the establishment of sister chromatid cohesion. *Nature Genetics*.

[21] Van den Berg DJ, Francke U (1993). Roberts syndrome: a review of 100 cases and a new rating system for severity. *American Journal of Medical Genetics*.

[22] Schüle B, Oviedo A, Johnston K, Pai S, Francke U (2005). Inactivating mutations in *ESCO2* cause SC phocomelia and Roberts syndrome: no phenotype-genotype correlation. *American Journal of Human Genetics*.

[23] Gordillo M, Vega H, Trainer AH (2008). The molecular mechanism underlying Roberts syndrome involves loss of ESCO2 acetyltransferase activity. *Human Molecular Genetics*.

[24] Vega H, Trainer AH, Gordillo M (2010). Phenotypic variability in 49 cases of *ESCO2* mutations, including novel missense and codon deletion in the acetyltransferase domain, correlates with *ESCO2* expression and establishes the clinical criteria for Roberts syndrome. *Journal of Medical Genetics*.

[25] Levitus M, Waisfisz Q, Godthelp BC (2005). The DNA helicase BRIP1 is defective in Fanconi anemia complementation group J. *Nature Genetics*.

[26] Meetei AR, Levitus M, Xue Y (2004). X-linked inheritance of Fanconi anemia complementation group B. *Nature Genetics*.

[27] Joenje H, Arwert F, Eriksson AW, de Koning H, Oostra AB (1981). Oxygen-dependence of chromosomal aberrations in Fanconi’s anaemia. *Nature*.

[28] Krantz ID, McCallum J, DeScipio C (2004). Cornelia de Lange syndrome is caused by mutations in *NIPBL*, the human homolog of *Drosophila melanogaster Nipped*-B. *Nature Genetics*.

[29] Tonkin ET, Wang T-J, Lisgo S, Bamshad MJ, Strachan T (2004). *NIPBL*, encoding a homolog of fungal Scc2-type sister chromatid cohesion proteins and fly Nipped-B, is mutated in Cornelia de Lange syndrome. *Nature Genetics*.

[30] Musio A, Selicorni A, Focarelli ML (2006). X-linked Cornelia de Lange syndrome owing to *SMC1L1* mutations. *Nature Genetics*.

[31] Deardorff MA, Kaur M, Yaeger D (2007). Mutations in cohesin complex members SMC3 and SMC1A cause a mild variant of Cornelia de Lange syndrome with predominant mental retardation. *American Journal of Human Genetics*.

